# Effects of the SGLT2 inhibitor canagliflozin on plasma biomarkers TNFR-1, TNFR-2 and KIM-1 in the CANVAS trial

**DOI:** 10.1007/s00125-021-05512-5

**Published:** 2021-08-20

**Authors:** Taha Sen, Jingwei Li, Brendon L. Neuen, Bruce Neal, Clare Arnott, Chirag R. Parikh, Steven G. Coca, Vlado Perkovic, Kenneth W. Mahaffey, Yshai Yavin, Norman Rosenthal, Michael K. Hansen, Hiddo J. L. Heerspink

**Affiliations:** 1 Department of Clinical Pharmacy and Pharmacology, University of Groningen, Groningen, the Netherlands; 2 The George Institute for Global Health, UNSW Sydney, Sydney, NSW Australia; 3 Johns Hopkins School of Medicine, Baltimore, MD USA; 4 Division of Nephrology, Icahn School of Medicine at Mount Sinai, New York, NY USA; 5 Stanford Center for Clinical Research, Department of Medicine, Stanford University School of Medicine, Stanford, CA USA; 6 Janssen Research & Development LLC, Spring House, PA USA

**Keywords:** Canagliflozin, Kidney and cardiovascular outcomes, KIM-1, SGLT2 inhibitor, TNFR-1, TNFR-2

## Abstract

**Aims/hypothesis:**

Higher plasma concentrations of tumour necrosis factor receptor (TNFR)-1, TNFR-2 and kidney injury molecule-1 (KIM-1) have been found to be associated with higher risk of kidney failure in individuals with type 2 diabetes in previous studies. Whether drugs can reduce these biomarkers is not well established. We measured these biomarkers in samples of the CANVAS study and examined the effect of the sodium–glucose cotransporter 2 inhibitor canagliflozin on these biomarkers and assessed whether the early change in these biomarkers predict cardiovascular and kidney outcomes in individuals with type 2 diabetes in the CANagliflozin cardioVascular Assessment Study (CANVAS).

**Methods:**

Biomarkers were measured with immunoassays (proprietary multiplex assay performed by RenalytixAI, New York, NY, USA) at baseline and years 1, 3 and 6. Mixed-effects models for repeated measures assessed the effect of canagliflozin vs placebo on the biomarkers. Associations of baseline levels and the early change (baseline to year 1) for each biomarker with the kidney outcome were assessed using multivariable-adjusted Cox regression.

**Results:**

In total, 3523/4330 (81.4%) of the CANVAS participants had available samples at baseline. Each doubling in baseline TNFR-1, TNFR-2 and KIM-1 was associated with a higher risk of kidney outcomes, with corresponding HRs of 3.7 (95% CI 2.3, 6.1; *p* < 0.01), 2.7 (95% CI 2.0, 3.6; *p* < 0.01) and 1.5 (95% CI 1.2, 1.8; *p* < 0.01), respectively. Canagliflozin reduced the level of the plasma biomarkers with differences in TNFR-1, TNFR-2 and KIM-1 between canagliflozin and placebo during follow-up of 2.8% (95% CI 3.4%, 1.3%; *p* < 0.01), 1.9% (95% CI 3.5%, 0.2%; *p* = 0.03) and 26.7% (95% CI 30.7%, 22.7%; *p* < 0.01), respectively. Within the canagliflozin treatment group, each 10% reduction in TNFR-1 and TNFR-2 at year 1 was associated with a lower risk of the kidney outcome (HR 0.8 [95% CI 0.7, 1.0; *p* = 0.02] and 0.9 [95% CI 0.9, 1.0; *p* < 0.01] respectively), independent of other patient characteristics. The baseline and 1 year change in biomarkers did not associate with cardiovascular or heart failure outcomes.

**Conclusions/interpretation:**

Canagliflozin decreased KIM-1 and modestly reduced TNFR-1 and TNFR-2 compared with placebo in individuals with type 2 diabetes in CANVAS. Early decreases in TNFR-1 and TNFR-2 during canagliflozin treatment were independently associated with a lower risk of kidney disease progression, suggesting that TNFR-1 and TNFR-2 have the potential to be pharmacodynamic markers of response to canagliflozin.

**Graphical abstract:**

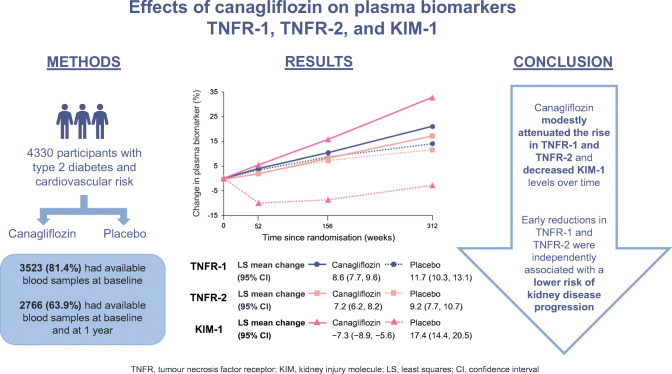

**Supplementary Information:**

The online version contains peer-reviewed but unedited supplementary material available at 10.1007/s00125-021-05512-5.

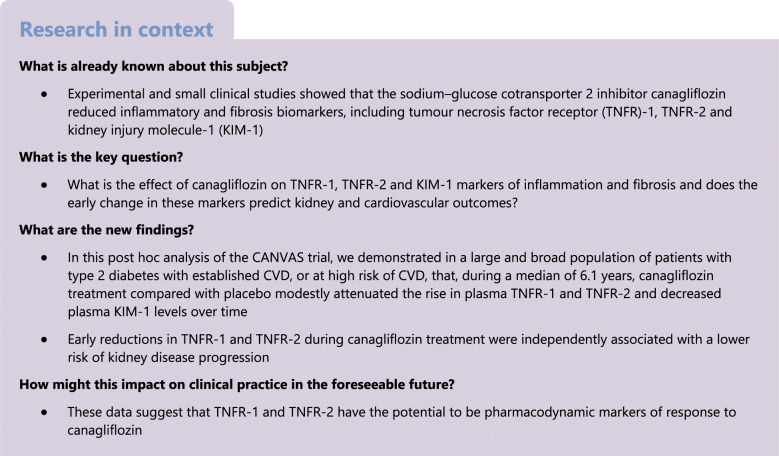



## Introduction

Approximately 40% of people with type 2 diabetes will develop diabetic kidney disease (DKD). As kidney function declines, the risk of kidney failure, cardiovascular morbidity and mortality increases [1]. Identifying individuals who will develop DKD and cardiovascular complications aids in tailoring therapies to those at highest risk of diabetes-related complications [2]. eGFR and urine albumin/creatinine ratio (UACR) are established biomarkers to identify high-risk patients [3]. Novel biomarkers that reflect the pathophysiological processes implicated in disease progression, such as inflammation, oxidative stress and fibrosis, may help in early risk stratification [4].

TNF-α is a key regulator of inflammation in individuals with DKD [5–9]. TNF-α can bind to its receptor (TNFR), which exists in multiple isoforms. Elevations of TNFR-1 or TNFR-2 are strong predictors of kidney failure [10, 11]. Kidney injury molecule-1 (KIM-1) is another plasma biomarker that has been shown to predict kidney failure [9]. This protein is located on the apical membrane of the proximal tubule and is released in the tubular lumen and taken up by the peri-tubular capillaries in circumstances of tubulointerstitial damage.

Sodium–glucose cotransporter 2 (SGLT2) inhibitors are a relatively new class of drug for the treatment of type 2 diabetes. Large cardiovascular and kidney outcome trials have shown that the SGLT2 inhibitor canagliflozin decreases the risks of adverse cardiovascular and kidney outcomes [12–15]. The precise mechanisms for these effects are incompletely understood. Small studies have suggested that canagliflozin may exert anti-inflammatory and anti-fibrotic effects that may in part explain their long-term kidney protective effects [16–18].

In this post hoc analysis of the CANagliflozin cardioVascular Assessment Study (CANVAS), we investigated whether baseline values of TNFR-1, TNFR-2 and KIM-1 predict kidney, cardiovascular and heart failure (HF) outcomes in patients with type 2 diabetes at high cardiovascular risk. Second, we assessed whether canagliflozin reduces the concentration of these biomarkers compared with placebo treatment. Finally, we assessed whether early changes in the biomarkers are associated with subsequent kidney, cardiovascular and HF outcomes in order to evaluate whether the biomarkers can potentially be used to monitor the efficacy of canagliflozin over time.

## Methods

### Participants and study design

The CANVAS Program consisted of two multicentre, double-blinded, placebo-controlled, randomised trials (CANVAS and CANVAS-R) to assess the effect of canagliflozin on primarily cardiovascular and secondarily kidney and safety outcomes in individuals with type 2 diabetes who had a history of CVD or multiple cardiovascular risk markers. The study design and the results were reported previously [14]. Blood and urine samples for exploratory biomarker research were stored during the CANVAS trial (and not the CANVAS-R trial). This study, therefore, only used samples provided from the CANVAS trial. All patients were offered the opportunity to participate in the exploratory biomarker research study. The CANVAS trial enrolled 4330 participants from 24 countries. Participants were randomly assigned using a central web-based response system in a 1:1:1 ratio to treatment with 100 mg canagliflozin, 300 mg canagliflozin or matching placebo. Participants assigned to treatment with canagliflozin or placebo were followed for a median of 6.1 years. All participants, care providers, trial staff and outcome assessors were blinded to treatment allocation for the duration of the study. All participants provided written informed consent. Separate informed consent for the collection of additional blood or urine samples for biomarker analysis was optional. The CANVAS trial was conducted according to the principles of the Declaration of Helsinki and was registered with clinicaltrials.gov (NCT01032629). The CANVAS trial was approved by an ethics committee at each participating site, and all participants provided written informed consent.

Eligible participants had type 2 diabetes with an HbA_1c_ level of ≥58 mmol/mol (7.0%) and ≤91 mmol/mol (10.5%), and were either 30 years or older with a history of symptomatic atherosclerotic CVD or at least 50 years of age with two or more risk factors for CVD. Risk factors for CVD were defined as a duration of diabetes of at least 10 years, systolic BP >140 mmHg, receiving >1 antihypertensive agent, current smoking, micro- or macroalbuminuria or an HDL-cholesterol level of <1 mmol/l. At inclusion, participants also needed to have an eGFR of >30 ml min^−1^ [1.73 m]^−2^ and meet other criteria for inclusion. A full list of these criteria is available in the appendix of the primary publication of the CANVAS Program data [14].

### Biomarker assessment

Blood samples for exploratory biomarker research were obtained at baseline and 52, 156 and 312 weeks after randomisation. For this study, plasma TNFR-1, TNFR-2 and KIM-1 were measured using the Mesoscale QuickPlex SQ 120 platform (Meso Scale Diagnostics [MSD], Rockville, MD, USA), which is a high-performance electrochemiluminescence immunoassay and was performed by RenalytixAI, New York, NY, USA. All biomarkers were measured between August 2019 and December 2019. Of the 3523 samples where TNFR-1, TNFR-2 and KIM-1 were measured, 469 samples were measured in duplicates, with the following mean (minimum, maximum) CV: TNFR-1: 2% (0%, 10%); TNFR-2: 2% (0%, 12%); and KIM-1: 3% (0%, 18%).

### Outcomes

The composite kidney outcome for this post hoc analysis was defined as a sustained 40% decline of eGFR, end-stage kidney disease defined as eGFR <15 ml min^−1^ [1.73 m]^−2^ or need for dialysis or kidney transplantation, or death related to kidney disease. The cardiovascular outcome for this study was defined as nonfatal myocardial infarction, nonfatal stroke or death due to cardiovascular cause. The HF outcome for this study was defined as hospitalisation for HF. These endpoints were adjudicated by a blinded adjudication committee using predefined and rigorous endpoint definitions [19].

### Statistical analysis

Continuous baseline variables with normal distributions were reported as means with SDs. Variables with skewed distributions were reported as median values with IQRs and were logarithmic transformed before analysis. Categorical variables were reported as percentages.

The HRs for the kidney, cardiovascular and HF outcomes for TNFR-1, TNFR-2 and KIM-1 categorised into quartiles or doubling of these biomarkers were estimated using multivariable Cox proportional hazard regression. Four consecutive models with different covariates were built to assess the impact of the covariates on the relationship between the plasma biomarkers and kidney or cardiovascular outcome. The first model included age, sex, race and treatment assignment as covariates. In the second model, the history of CVD, HbA_1c_, current smoking status, systolic and diastolic BP, BMI and LDL-cholesterol were added. Baseline eGFR was added in the third model. Lastly, log transformed UACR was added in the fourth model. The analyses with all the mentioned models were also performed in subgroups defined by randomised treatment and baseline age, sex, eGFR, UACR and CVD history in the fully adjusted model to assess effect modification by these variables. We assessed C statistics, net reclassification improvement (NRI) and integrated discrimination improvement (IDI) to assess the discriminative ability of the biomarkers.

Cox proportional hazard regression models were fitted to explore whether baseline TNFR-1, TNFR-2 or KIM-1 modified the treatment effect of canagliflozin vs placebo on kidney, cardiovascular and HF outcomes. Tests for heterogeneity were performed by adding interaction terms between the plasma biomarkers, fitted as a categorical variable, and randomised treatment assignment to the relevant Cox models.

The effect of canagliflozin vs placebo on TNFR-1, TNFR-2 and KIM-1 concentrations over time was assessed by calculating the difference of percentage change in the geometric mean of the biomarker between treatment arms using mixed effects models. The model included treatment allocation and visit-time as factors and an interaction term between treatment allocation and visit-time. Visits were included as repeated units from the same patient. The model was also adjusted for the baseline biomarker value and interaction term between visit and baseline biomarker value. The variance–covariance matrix was assumed to be unstructured, i.e. purely data dependent. All participants and all data points were included. No patients were excluded because of missing data and no imputation was done for missing data. The between-group geometric mean change was derived by 100 × (*e*^LSM^ − 1).

Associations between the 1 year change in TNFR-1, TNFR-2 and KIM-1 from baseline were assessed using Cox proportional hazard regression adopting a landmark approach. All kidney, cardiovascular and HF endpoints that occurred in the first year were excluded from the analysis. Quartiles of the 1 year change in each biomarker were fitted in a Cox proportional hazard regression model. The models were adjusted using the same covariates as described above for the association between baseline marker and kidney, cardiovascular or HF outcome, as well as 1 year change in log transformed UACR, eGFR, systolic BP, BMI and HbA_1c_. The first model included baseline biomarker (TNFR-1, TNFR-2 or KIM-1), age, sex and randomised treatment as covariates. eGFR was added in the second model and replaced for UACR in the third model. eGFR and UACR were both added in the fourth model. In the fifth model, history of CVD, current smoking status, HbA_1c_, systolic and diastolic BP, BMI, LDL-cholesterol, and the change in systolic BP, BMI and HbA_1c_ from baseline to year 1 were added.

For each outcome, we also provided a descriptive assessment of the percentage of the randomised treatment effect removed with adjustment for change in plasma biomarker levels from baseline to year 1, as was done previously in the CANVAS trial [20]. For each outcome, the percentage of the treatment effect explained was expressed using the equation: 100% × ([HR − HRadjusted]/[HR − 1]). Results were deemed significant when *p* < 0.05. All analyses were performed in SAS version 9.4 (SAS Institute, Cary, NC, USA) and Stata version 16.1 (StataCorp, College Station, TX, USA).

## Results

### Study population

**Fig. 1:**
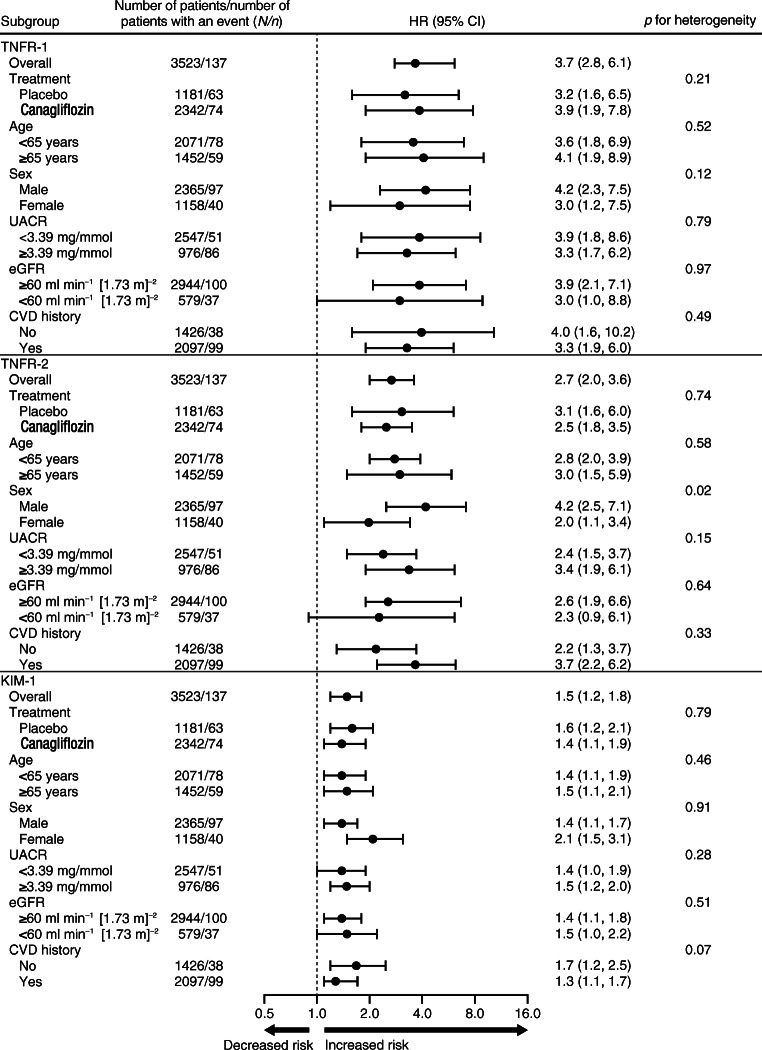
Associations of the doubling in each baseline biomarker with the kidney outcome in subgroups defined by baseline patient characteristics. Models are adjusted for the following covariates: age, sex, race and randomised treatment; history of CVD, HbA_1c_, current smoking, systolic and diastolic BP, BMI and LDL-cholesterol; baseline eGFR; and log transformed baseline UACR

Of 4330 participants in the CANVAS trials, 3523 (81.4%) had available blood samples at baseline and 2766 (63.9%) had blood samples available at baseline and at 1 year. For the analysis on the association between 1 year changes in biomarkers and subsequent kidney, cardiovascular and HF outcomes, 6, 55 and 8 participants of the 2766 were excluded, respectively, since they experienced the kidney, cardiovascular or HF outcome before year 1 (Electronic supplementary material [ESM] Fig. 1).

Baseline patient characteristics of 3523 participants are shown in Table 1. Participants had a mean age of 62.8 years, 67.1% were male, 59.5% had a history of CVD, the mean duration of diabetes mellitus was 13.5 years, the mean HbA_1c_ was 66 mmol/mol (8.2%) and the mean eGFR was 77.0 ml min^−1^ [1.73 m]^−2^. Median levels of TNFR-1, TNFR-2 and KIM-1 were 2577 pg/ml, 9682 pg/ml and 110 pg/ml, respectively. All characteristics were well balanced in participants randomised to treatment with canagliflozin compared with placebo and were comparable to the baseline characteristics of the overall trial reported previously [14].

**Table 1:** Baseline characteristics of the total, placebo and canagliflozin-treated population with baseline samples analysed

Characteristic	Total *N*=3523	Placebo *N*=1181	Canagliflozin *N*=2342
Age, years	62.8 (7.9)	62.5 (7.8)	62.9 (7.9)
Male sex, *n* (%)	2365 (67.1)	795 (67.3)	1570 (67.0)
Current smoker, *n* (%)	642 (18.2)	241 (20.4)	401 (17.1)
History of HF, *n* (%)	469 (13.3)	173 (14.7)	296 (12.6)
Duration of diabetes, years	13.5 (7.5)	13.4 (7.6)	13.6 (7.5)
History of CVD, *n* (%)	2097 (59.5)	699 (59.2)	1398 (59.7)
BMI, kg/m^2^	32.7 (6.1)	32.6 (6.2)	32.7 (6.1)
Systolic BP, mmHg	136.7 (15.8)	137.3 (15.7)	136.4 (15.9)
Diastolic BP, mmHg	77.6 (9.7)	78.1 (9.8)	77.3 (9.7)
HbA_1c_			
mmol/mol	66 (9.9)	66 (9.8)	66 (9.9)
%	8.17 (0.91)	8.16 (0.90)	8.17 (0.91)
eGFR, ml min^−1^ [1.73 m]^−2^	77.0 (18.8)	76.8 (18.9)	77.0 (18.7)
eGFR <60, *n* (%)	579 (16.4)	207 (17.5)	372 (15.9)
eGFR ≥60, *n* (%)	2944 (83.6)	974 (82.5)	1970 (84.1)
UACR, mg/mmol	1.3 (0.7–4.0)	1.3 (0.7–4.2)	1.3 (0.7–3.9)
Normoalbuminuria, *n* (%)	2547 (72.3)	844 (71.5)	1703 (72.7)
Microalbuminuria, *n* (%)	775 (22.0)	256 (21.7)	519 (22.2)
Macroalbuminuria, *n* (%)	201 (5.7)	81 (6.9)	120 (5.1)
TNFR-1, pg/ml	2577 (2126–3158)	2570 (2133–3114)	2580 (2123–3170)
TNFR-2, pg/ml	9682 (7827–11,971)	9556 (7800–12,038)	9732 (7843–11,934)
KIM-1, pg/ml	110 (72–175)	112 (73–178)	108 (71–171)

Continuous variables are reported as mean (SD) or median (IQR); categorical variables are reported as *n* (%)

### Association between baseline plasma biomarkers with kidney and cardiovascular outcomes

Participants were followed for a median duration of 6.1 (25th to 75th percentile: 5.8 to 6.4) years, during which 137 (3.9%), 548 (15.6%) and 128 (3.6%) participants experienced a kidney, cardiovascular or HF outcome, respectively. Pearson correlation coefficients showed generally weak correlations between the baseline values of the plasma biomarkers and cardiovascular risk markers except for baseline eGFR and UACR (ESM Fig. 2). In multivariable analyses adjusting for patient demographics, the plasma biomarkers were statistically significantly associated with the kidney outcome (Table 2). Further stepwise adjustment for cardiovascular risk markers revealed a modest attenuation of the HR only in the final model when the associations were adjusted for baseline UACR (Table 2). In the fully adjusted model, each doubling in TNFR-1, TNFR-2 or KIM-1 was significantly associated with an increased risk of kidney outcomes, with adjusted HRs of 3.7 (95% CI 2.3, 6.1; *p* < 0.01), 2.7 (95% CI 2.0, 3.6; *p* < 0.01) and 1.5 (95% CI 1.2, 1.8; *p* < 0.01), respectively (Table 2). We found a more modest association between TNFR-1 and cardiovascular outcomes (HR per doubling 1.3 [95% CI 1.0, 1.6]; *p* = 0.049). There was no statistically significant association between TNFR-2 and KIM-1 with cardiovascular outcomes; the corresponding HRs were 1.2 (95% CI 1.0, 1.5; *p* = 0.07) and 1.0 (95% CI 0.9, 1.1; *p* = 0.56), respectively (ESM Table 1). The plasma biomarkers also did not associate with hospitalisation for HF during follow-up (ESM Table 2). Results of the association of the doubling in biomarker with outcomes in the fully adjusted model were generally similar in subgroup analyses defined by baseline patient characteristics for the kidney (Fig. 1), cardiovascular (ESM Fig. 3) and HF outcome (ESM Fig. 4), although the association between TNFR-2 and kidney outcomes may vary by sex. When biomarkers were modelled as categorical variables, the relative risk of the kidney outcome was significantly higher in the highest vs lowest quartile of TNFR-1 and TNFR-2 (Table 2). Adding the biomarkers to clinical variables significantly improved the C statistic, NRI and IDI (ESM Table 3). Analyses with time-dependent C statistics showed good prognostic performance for the kidney outcome (ESM Table 4).

**Table 2:** Associations of baseline TNFR-1, TNFR-2 and KIM-1 with the composite kidney outcome adjusting for covariates

Biomarker	Model 1		Model 2		Model 3		Model 4	
	HR (95% CI)	*p* value	HR (95% CI)	*p* value	HR (95% CI)	*p* value	HR (95% CI)	*p* value
TNFR-1								
Per doubling	5.4 (3.8, 7.6)	<0.01	4.9 (3.4, 7.1)	<0.01	8.3 (5.2, 13.2)	<0.01	3.7 (2.3, 6.1)	<0.01
Quartile 1	(reference)		(reference)		(reference)		(reference)	
Quartile 2	2.2 (1.0, 4.6)	0.04	1.9 (0.9, 4.1)	0.08	2.0 (1.0, 4.3)	0.06	2.0 (0.9, 4.2)	0.08
Quartile 3	3.7 (1.8, 7.5)	<0.01	3.2 (1.6, 6.6)	<0.01	3.6 (1.8, 7.5)	<0.01	2.8 (1.4, 5.8)	0.05
Quartile 4	7.8 (4.0, 15.4)	<0.01	6.4 (3.2, 12.8)	<0.01	7.9 (3.8, 16.3)	<0.01	4.2 (2.0, 8.9)	<0.01
TNFR-2								
Per doubling	2.9 (2.4, 3.4)	<0.01	3.1 (2.5, 3.8)	<0.01	3.1 (2.5, 3.9)	<0.01	2.7 (2.0, 3.6)	<0.01
Quartile 1	(reference)		(reference)		(reference)		(reference)	
Quartile 2	2.0 (1.0, 4.2)	0.06	1.9 (0.9, 3.9)	0.09	2.0 (0.9, 4.0)	0.07	1.9 (0.9, 3.8)	0.10
Quartile 3	2.4 (1.2, 4.9)	0.02	2.1 (1.0, 4.4)	0.04	2.3 (1.1, 4.8)	0.02	1.9 (0.9, 3.9)	0.08
Quartile 4	8.2 (4.3, 15.5)	<0.01	7.1 (3.7, 13.7)	<0.01	8.4 (4.3, 16.5)	<0.01	4.8 (2.4, 9.6)	<0.01
KIM-1								
Per doubling	2.3 (2.0, 2.6)	<0.01	2.1 (1.8, 2.5)	<0.01	2.2 (1.9, 2.6)	<0.01	1.5 (1.2, 1.8)	<0.01
Quartile 1	(reference)		(reference)		(reference)		(reference)	
Quartile 2	1.0 (0.5, 2.0)	0.95	1.0 (0.5, 1.9)	0.90	1.0 (0.5, 1.9)	0.90	0.8 (0.4, 1.5)	0.45
Quartile 3	1.5 (0.8, 2.8)	0.20	1.4 (0.7, 2.6)	0.31	1.4 (0.7, 2.6)	0.31	0.9 (0.5, 1.7)	0.80
Quartile 4	5.2 (3.0, 8.8)	<0.01	4.3 (2.5, 7.4)	<0.01	4.2 (2.4, 7.4)	<0.01	1.6 (0.9, 3.0)	0.11

Models are adjusted for the following covariates. Model 1: age, sex, race and randomised treatment. Model 2: covariates of model 1 + history of CVD, HbA_1c_, current smoking, systolic and diastolic BP, BMI and LDL-cholesterol. Model 3: covariates of model 2 + baseline eGFR. Model 4: covariates of model 3 + log transformed baseline UACR

### Effect of canagliflozin on kidney and cardiovascular outcomes by baseline plasma biomarker levels

**Fig. 2:**
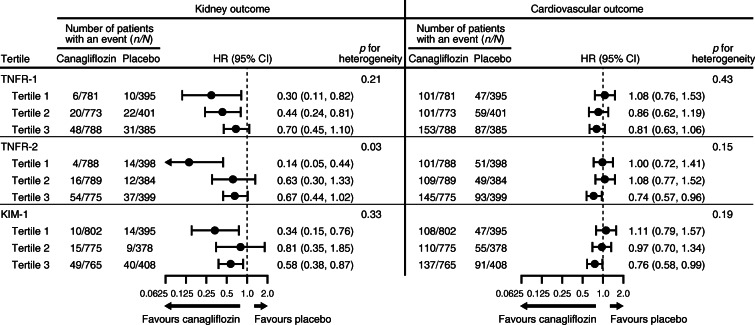
Forest plot of the effect of canagliflozin on kidney and cardiovascular outcomes by baseline TNFR-1, TNFR-2 and KIM-1 divided in tertiles. When the biomarkers were fitted as continuous variables, the *p* values for heterogeneity for the kidney and cardiovascular outcome were: TNFR-1: 0.13 and 0.39, respectively; TNFR-2: 0.18 and 0.09, respectively; KIM-1: 0.77 and 0.07, respectively

In all participants with baseline biomarkers, the composite kidney outcome occurred less frequently in the canagliflozin group compared with the placebo group (HR 0.56; 95% CI 0.40, 0.78; *p* < 0.01). The HR associated with canagliflozin for the cardiovascular outcome was 0.90 (95% CI 0.76, 1.07; *p* = 0.25). There was no evidence that the effects of canagliflozin on the kidney, cardiovascular and HF outcomes varied by the baseline level of the plasma biomarkers (all *p* values for heterogeneity >0.15), except for TNFR-2 with the kidney outcome (*p* value for heterogeneity = 0.03). However, when the biomarkers were fitted as continuous variables, there was no evidence that the effect of canagliflozin was different on the kidney, cardiovascular and HF outcome (all *p* values for heterogeneity >0.07; Fig. 2 and ESM Fig. 5).

### Effect of canagliflozin on plasma biomarkers

**Fig. 3:**
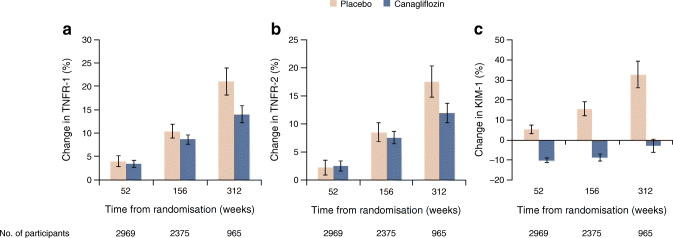
Change in plasma TNFR-1, TNFR-2 and KIM-1 in canagliflozin vs placebo-treated participants over time. (**a**) Percentage change from baseline in TNFR-1; LSM change placebo 11.7% (95% CI 10.3, 13.1); LSM change canagliflozin 8.6% (95% CI 7.7, 9.6). (**b**) Percentage change from baseline in TNFR-2; LSM change placebo 9.2% (95% CI 7.7, 10.7); LSM change canagliflozin 7.2% (95% CI 6.2, 8.2). (**c**) Percentage change in KIM-1; LSM change placebo 17.4% (95% CI 14.4, 20.5); LSM change canagliflozin –7.3% (95% CI –8.9, –5.6). LSM, least square mean

The concentrations of TNFR-1 and TNFR-2 increased over time in the placebo group (Fig. 3). During follow-up, canagliflozin attenuated the increase in TNFR-1 and TNFR-2 compared with placebo, resulting in a modest least square mean difference in TNFR-1 of 2.8% (95% CI 1.3%, 3.4%; *p* < 0.01) and in TNFR-2 of 1.9% (95% CI 0.2%, 3.5%; *p* = 0.03) (Fig. 3). At year 1, canagliflozin reduced plasma KIM-1 levels by 26.7% (95% CI 22.7%, 30.7%; *p* < 0.01) (Fig. 3) compared with placebo. This effect persisted over time until the end of follow-up. The effect of canagliflozin compared with placebo on the plasma biomarkers was generally consistent in subgroups defined by baseline UACR <3.39 or ≥3.39 mg/mmol or eGFR <60 or ≥60 ml min^−1^ [1.73 m]^−2^. A statistically significant interaction was observed for the effect of canagliflozin on TNFR-1 in UACR subgroups and for TNFR-2 in eGFR subgroups, but absolute differences between subgroups were modest (Table 3).

**Table 3:** Changes in the biomarkers TNFR-1, TNFR-2 and KIM-1 in the canagliflozin-treated and placebo group from baseline to year 1 by subgroup

Biomarker	Baseline biomarker in canagliflozin, pg/ml	Baseline biomarker in placebo, pg/ml	Canagliflozin change, % (95% CI)	Placebo change, % (95% CI)	Placebo corrected effect canagliflozin, % (95% CI)	*p* interaction
TNFR-1						
UACR, mg/mmol						0.049
<3.39	2456	2488	7.1 (6.2, 8.0)	9.6 (8.2, 11.1)	−2.3 (−3.8, −0.8)	
≥3.39	2913	2838	12.5 (10.8, 14.1)	17.3 (14.8, 19.8)	−4.1 (−6.6, −1.6)	
eGFR, ml min^−1^ [1.73 m]^−2^						0.471
<60	3505	3288	4.7 (3.9, 5.6)	7.8 (6.5, 9.1)	−2.4 (−4.6, −0.1)	
≥60	2446	2457	20.4 (18.7, 22.0)	23.3 (21.0, 25.7)	−2.8 (−4.2, −1.4)	
TNFR-2						
UACR, mg/mmol						0.507
<3.39	9312	9251	5.4 (4.4, 6.4)	7.9 (6.3, 9.5)	−2.3 (−4.0, −0.6)	
≥3.39	10,836	10,664	11.6 (9.7, 13.5)	13.2 (10.5, 16.0)	−1.5 (−4.3, 1.5)	
eGFR, ml min^−1^ [1.73 m]^−2^						0.025
<60	12,669	12,495	3.4 (2.4, 4.3)	6.0 (4.6, 7.5)	−0.2 (−2.8, 2.5)	
≥60	9262	9137	18.6 (16.8, 20.5)	18.8 (16.2, 21.5)	−2.5 (−4.1, −0.9)	
KIM-1						
UACR, mg/mmol						0.132
<3.39	94	98	−8.6 (−10.4, −7.1)	13.6 (10.6, 16.8)	−19.7 (−22.3, −17.1)	
≥3.39	162	148	−3.6 (−6.6, −0.4)	28.5 (22.8, 34.4)	−24.9 (−28.9, −20.7)	
eGFR, ml min^−1^ [1.73 m]^−2^						
<60	143	141	−9.1 (−10.7, −7.5)	12.8 (10.0, 15.8)	−25.4 (−29.0, −21.6)	0.573
≥60	103	104	−2.5 (−5.2, 0.4)	30.7 (25.5, 36.2)	−19.5 (−21.9, −16.9)	

### Associations between changes in biomarkers and outcomes

Among the 2766 participants with baseline and year 1 samples available, median concentrations of TNFR-1, TNFR-2 and KIM-1 were 2599 pg/ml, 9691 pg/ml and 106 pg/ml, respectively, at baseline. We examined the association of the change in the biomarkers from baseline to year 1 with subsequent kidney or cardiovascular outcomes. Among the 2760 participants who were included in this analysis, 110 (4.0%) experienced the kidney outcome after 1 year. Pearson correlation coefficients showed generally weak correlations between the 1 year change in the plasma biomarkers with cardiovascular risk markers except for 1 year changes in eGFR and UACR (ESM Fig. 6). In multivariable analysis after adjustment for all covariates, each 10% reduction in TNFR-1 and TNFR-2 was independently associated with a lower risk of the kidney outcome with corresponding HRs of 0.8 (95% CI 0.7, 1.0; *p* = 0.02) and 0.9 (95% CI 0.9, 1.0; *p* < 0.01), respectively (model 5, Table 4). In contrast, changes in KIM-1 from baseline to year 1 did not associate with kidney outcomes in the fully adjusted multivariable model with a corresponding HR of 1.0 (95% CI 0.9, 1.0; *p* = 0.44) for each 10% reduction in KIM-1 (model 5, Table 4). When TNFR-1 and TNFR-2 were analysed as categorical variables in the fully adjusted model compared with the reference group with little change in TNFR, the highest quartiles of TNFR-1 and TNFR-2 were associated with a two- to threefold increased risk of the kidney outcome (Table 4).

**Table 4:** Associations of the quartiles of each change in TNFR-1, TNFR-2 and KIM-1 from baseline to year 1 with the composite kidney outcome in five different models

Biomarker		Model 1		Model 2		Model 3		Model 4		Model 5	
	Median change	HR (95% CI)	*p* value	HR (95% CI)	*p* value	HR (95% CI)	*p* value	HR (95% CI)	*p* value	HR (95% CI)	*p* value
TNFR-1											
Per 10% reduction		0.7 (0.6, 0.8)	<0.01	0.8 (0.7, 0.9)	<0.01	0.8 (0.7, 0.9)	<0.01	0.9 (0.7, 1.0)	0.04	0.8 (0.7, 1.0)	0.02
Quartile 1	−11.7	0.8 (0.4, 1.5)	0.50	0.9 (0.5, 1.7)	0.77	0.9 (0.5, 1.6)	0.64	1.0 (0.5, 1.9)	0.98	0.9 (0.5, 1.8)	0.83
Quartile 2	−0.6	(reference)		(reference)		(reference)		(reference)		(reference)	
Quartile 3	8.2	1.4 (0.7, 2.5)	0.31	1.4 (0.7, 2.5)	0.32	1.3 (0.7, 2.4)	0.39	1.3 (0.7, 2.5)	0.35	1.4 (0.7, 2.6)	0.30
Quartile 4	21.1	3.1 (1.8, 5.2)	<0.01	2.5 (1.5, 4.4)	<0.01	2.4 (1.4, 4.1)	<0.01	2.1 (1.2, 3.6)	0.01	2.2 (1.2, 3.8)	0.01
TNFR-2											
Per 10% reduction		0.9 (0.9, 1.0)	<0.01	0.9 (0.9, 1.0)	0.02	0.9 (0.9, 0.9)	<0.01	0.9 (0.9, 1.0)	<0.01	0.9 (0.9, 1.0)	<0.01
Quartile 1	−12.8	1.1 (0.6, 2.2)	0.68	1.4 (0.7, 2.7)	0.36	1.0 (0.5, 2.0)	0.90	1.3 (0.7, 2.6)	0.43	1.2 (0.6, 2.3)	0.67
Quartile 2	−2.2	(reference)		(reference)		(reference)		(reference)		(reference)	
Quartile 3	6.2	1.8 (1.0, 3.3)	0.07	1.9 (1.0, 3.5)	0.05	1.9 (1.0, 3.5)	0.04	2.2 (1.2, 4.1)	0.01	2.3 (1.2, 4.3)	0.01
Quartile 4	20.0	3.2 (1.8, 5.7)	<0.01	2.7 (1.5, 4.8)	<0.01	2.9 (1.6, 5.2)	<0.01	3.0 (1.7, 5.5)	<0.01	2.9 (1.6, 5.3)	<0.01
KIM-1											
Per 10% reduction		0.9 (0.9, 1.0)	<0.01	0.9 (0.9, 1.0)	0.01	0.9 (0.9, 1.0)	0.02	1.0 (0.8, 1.0)	0.29	1.0 (0.9, 1.0)	0.44
Quartile 1	−34.0	0.9 (0.5, 1.6)	0.79	0.9 (0.5, 1.6)	0.72	0.9 (0.5, 1.7)	0.84	1.0 (0.6, 1.7)	0.95	1.1 (0.6, 1.9)	0.74
Quartile 2	−13.3	(reference)		(reference)		(reference)		(reference)		(reference)	
Quartile 3	2.9	0.9 (0.5, 1.7)	0.73	0.9 (0.5, 1.7)	0.78	0.8 (0.4, 1.4)	0.38	0.8 (0.4, 1.4)	0.38	0.7 (0.4, 1.3)	0.29
Quartile 4	33.1	1.9 (1.1, 3.2)	0.02	1.6 (0.9, 2.7)	0.10	1.5 (0.9, 2.6)	0.16	1.1 (0.6, 2.0)	0.65	1.0 (0.6, 1.8)	0.91

Models are adjusted for the following covariates. Model 1: baseline biomarker (TNFR-1, TNFR-2 or KIM-1), age, sex, race and randomised treatment. Model 2: covariates of model 1 + change in eGFR from baseline to year 1 and baseline eGFR. Model 3: covariates of model 1 + change in UACR from baseline to year 1 and baseline UACR. Model 4: covariates of model 1 + change in eGFR and UACR from baseline to year 1 and baseline eGFR and UACR. Model 5: covariates of model 1 + history of CVD, current smoking, HbA_1c_, systolic and diastolic BP, BMI, LDL-cholesterol, eGFR, baseline UACR, and change in eGFR, UACR, systolic BP, BMI and HbA_1c_ from baseline to year 1

In regard to cardiovascular outcomes and hospitalisation for HF after 1 year, 349 (12.9%) and 95 (3.4%) participants experienced these outcomes during follow-up, respectively. In continuous or quartile analyses, we did not find an association between early changes in the plasma biomarkers and cardiovascular outcomes or HF outcomes (ESM Table 5 and ESM Table 6). The association between 1 year changes from baseline in TNFR-1 and TNFR-2 and kidney outcomes were consistent in the placebo and canagliflozin groups (p for interaction 0.60 and 0.20, respectively; ESM Table 7).

Analyses of the proportion of treatment effects on kidney outcomes explained by the change in the plasma biomarkers showed that reductions in TNFR-1, TNFR-2 and KIM-1 with canagliflozin explained −6.8%, −13.6% and 4.5% for the kidney outcome, respectively (ESM Table 8).

## Discussion

Previous studies have established a strong association between plasma TNFR-1, TNFR-2 and KIM-1 and kidney outcomes in individuals with type 2 diabetes with and without chronic kidney disease [5, 10, 21]. This study confirms and extends these findings by demonstrating that, among participants with type 2 diabetes and established CVD or at high cardiovascular risk participating in the CANVAS trial, TNFR-1, TNFR-2 and KIM-1 predict adverse kidney outcomes. In addition, we demonstrated that the SGLT2 inhibitor canagliflozin attenuates the increase in TNFR-1 and TNFR-2 and decreases KIM-1 compared with placebo. Furthermore, increases in TNFR-1 and TNFR-2 after 1 year were associated with increased risk of subsequent kidney outcomes independent of baseline and early changes in other markers of cardiovascular or kidney disease progression, including UACR and eGFR. In contrast, there were no associations between baseline or change in plasma biomarkers with cardiovascular or HF outcomes.

Most previous studies demonstrating a strong positive association between the plasma biomarkers and kidney outcomes were relatively small single-centre studies and were conducted in relatively homogeneous populations. We confirm the prognostic performance of the TNFR biomarkers and KIM-1 for kidney outcomes and extend these initial findings to a large global heterogeneous population of various ethnicities treated according to contemporary guidelines. The lack of a significant association of the plasma biomarkers with cardiovascular outcomes suggests a kidney-specific relationship in individuals with type 2 diabetes with established or at high risk of CVD. In contrast, previous studies have shown significant associations of TNFR-1 and TNFR-2 with cardiovascular outcomes in individuals with type 2 diabetes mellitus [22]. In the meta-analysis of the previous studies, HF, ischaemic heart disease and peripheral vascular disease were included in the definition of the cardiovascular outcome. Differences in the specific definitions of the cardiovascular outcome and the rigorous adjudication of cardiovascular outcomes in the CANVAS trial may have contributed to these different findings. Importantly, these studies did not adjust their models for UACR. We adjusted for UACR and observed that adjustment for UACR markedly attenuated the strength of the association between TNFR-1 and TNFR-2 with the cardiovascular outcome. We also note that the majority of participants in the CANVAS trial had relatively preserved kidney function whereas other studies included participants with chronic kidney disease. These differences in patient populations could also explain these contrasting findings. Furthermore, we did not find an association between TNFR-1, TNFR-2 or KIM-1 and hospitalisation for HF. Other studies in patients with atherosclerosis or chronic HF also did not find an association between these biomarkers and incidence of HF [23, 24].

The initiation and progression of kidney disease in individuals with type 2 diabetes is heterogeneous and involves multiple pathophysiological pathways, including inflammation and fibrosis [4]. The TNF signalling pathways involve cytokines produced by immune cells. TNF-α, the principle cytokine, can bind to the transmembrane TNFR-1, located in glomeruli and endothelial cells, and TNFR-2, expressed transcriptionally in tubular epithelial cells of the kidney [10, 11]. The ubiquitous presence of TNFR-1 and TNFR-2 in the kidney may explain the strong association between the plasma concentration of these proteins and kidney outcomes. Other pivotal pathways involved in the progression of DKD are thought to reflect fibrosis. KIM-1 is apically expressed on the membrane of the proximal tubule and is shed after tubular injury and is thought to promote fibrosis. KIM-1 can be found in circulation as a result of possible increased transepithelial permeability or loss of epithelial cell polarity with basolateral membrane expression [5, 25]. The associations of plasma KIM-1 levels with kidney outcomes in our study, in whom the vast majority of patients had chronic kidney disease stage 1 or 2, suggest that fibrosis is involved in kidney disease progression even when kidney function is relatively preserved.

Canagliflozin reduced kidney and cardiovascular outcomes consistently regardless of the baseline concentration of the plasma biomarkers. However, since the absolute risk for kidney outcomes was higher at higher levels of each plasma biomarker, the absolute risk reductions for kidney and cardiovascular outcomes were higher in the highest tertile of the plasma biomarker, which supports the initiation of canagliflozin, particularly in these high-risk participants.

Overall, we observed in this study that canagliflozin modestly attenuates the elevation of both TNFR-1 and TNFR-2 and decreases KIM-1 levels compared with placebo. This effect persisted in various subgroups, including subgroups of participants with lower baseline eGFR and higher degrees of UACR. The modest attenuation in the increase in TNFR-1 has been observed in a prior study with canagliflozin [18]. However, the magnitude of the effect was smaller in the present study. Nevertheless, both studies support a potential role for canagliflozin in attenuating inflammatory processes in individuals with type 2 diabetes. Baricitinib, a Janus kinase inhibitor, is the only other drug that has been shown to lower TNFR levels in patients with type 2 diabetes and chronic kidney disease [26]. Canagliflozin also significantly reduced systemic KIM-1 levels. Previous studies have shown that SGLT2 inhibitors decrease urinary KIM-1 levels, suggesting an attenuation of interstitial fibrosis. This could explain the kidney specific association of KIM-1 and the lack of association with the cardiovascular outcome, although it is interesting that KIM-1 predicts cardiovascular outcomes in those with CVD, which warrants confirmation in an independent study [27]. The sustained reduction in plasma KIM-1 levels with canagliflozin in our study confirms these initial findings and extends them to a much larger and broader population.

The 1 year change in TNFR-1 and TNFR-2 was associated with kidney outcomes such that patients with a decrease in these plasma biomarkers during canagliflozin treatment were at decreased risk of kidney outcomes, while the reverse was true for those with an increase in the biomarkers. These data suggest that both TNFR-1 and TNFR-2 have the potential to be markers of early response to treatment with canagliflozin for the prevention of kidney outcomes, but they do not imply that the benefit of canagliflozin on kidney outcomes is mediated through reductions in TNFR-1 or TNFR-2. Canagliflozin reduced KIM-1 levels, but the association between changes in KIM-1 and kidney outcome lost statistical significance when adjusted for baseline and 1 year changes in UACR and eGFR.

This study has limitations. We performed observational analyses that cannot be used to infer causality. Therefore, the association between 1 year change in TNFR-1 and TNFR-2 with kidney outcomes should not be interpreted that reducing inflammation with canagliflozin prevents kidney outcomes. Our findings that 1 year changes in TNFR-1, TNFR-2 and KIM-1 did not explain the canagliflozin treatment effects on kidney outcomes suggest that although changes in these markers are predictive, they are not on the causal pathway. Second, canagliflozin lowers HbA_1c_ and the reduction in TNFR and KIM-1 may be explained by the improvement in glycaemic control. However, a previous study comparing canagliflozin with an active control, glimepiride, also reported reductions in TNFR without clear differences in glycaemic control, supporting a direct anti-inflammatory effect of canagliflozin [18]. Finally, although we enrolled a broad global population, the findings of this study can only be applied to patients who share the characteristics of the CANVAS cohort.

In conclusion, we confirm the prognostic association of inflammatory markers TNFR-1, TNFR-2 and KIM-1 with kidney outcomes in individuals with type 2 diabetes and established CVD or at high cardiovascular risk. In addition, treatment with canagliflozin attenuated elevations of TNFR-1 and TNFR-2 over time and reduced KIM-1 concentrations. This effect was consistent in various patient subgroups. One year changes in TNFR-1 and TNFR-2 correlated with kidney outcomes, suggesting that both inflammatory markers may be used as pharmacodynamic response markers to canagliflozin.

## Supplementary information

ESM (PDF 523 kb)

## Data Availability

Data from the CANVAS trial is available in the public domain via the Yale University Open Data Access Project (YODA; http://yoda.yale.edu).

## Abbreviations

CANVAS: CANagliflozin cardioVascular Assessment Study
DKD: Diabetic kidney disease
HF: Heart failure
IDI: Integrated discrimination improvement
KIM-1: Kidney injury molecule-1
NRI: Net reclassification improvement
SGLT2: Sodium–glucose cotransporter 2
TNFR: Tumour necrosis factor receptor
UACR: Urine albumin/creatinine ratio
