# Supplementary material for: Effects of the SGLT2 inhibitor canagliflozin on plasma biomarkers TNFR-1, TNFR-2 and KIM-1 in the CANVAS trial
Source: Diabetologia. 2021 Aug 20;64(10):2147–58. doi: 10.1007/s00125-021-05512-5 (PMC8423682; doi:10.1007/s00125-021-05512-5)
Supplement: Supplementary file 1 — (PDF 523 kb) [file 125_2021_5512_MOESM1_ESM.pdf]

Electronic Supplementary Material (ESM) to: Effects of the SGLT2 Inhibitor Canagliflozin on Plasma Biomarkers TNFR-1, TNFR-2, and KIM-1 in the CANVAS Trial

Sen T. et. al

ESM Table 1. Associations of the quartiles and the doubling of TNFR-1, TNFR-2, and KIM-1 with the composite cardiovascular outcome

Biomarker		Model 1		Model 2		Model 3		Model 4	
		**HR (95% CI)**	***p* value**	**HR (95% CI)**	***p* value**	**HR (95% CI)**	***p* value**	**HR (95% CI)**	***p* value**
**TNFR-1**									
	Per doubling	1.7 (1.4, 2.1)	<0.01	1.6 (1.3, 1.9)	<0.01	1.5 (1.2, 1.9)	<0.01	1.3 (1.0, 1.6)	0.05
	Quartile 1	(reference)		(reference)		(reference)		(reference)	
	Quartile 2	1.1 (0.9, 1.4)	0.42	1.0 (0.8, 1.3)	0.78	1.0 (0.8, 1.3)	0.96	1.0 (0.8, 1.3)	0.95
	Quartile 3	1.2 (0.9, 1.5)	0.20	1.1 (0.9, 1.4)	0.43	1.0 (0.8, 1.4)	0.74	1.0 (0.8, 1.3)	0.98
	Quartile 4	1.6 (1.3, 2.1)	<0.01	1.4 (1.1, 1.9)	0.01	1.3 (1.0, 1.7)	0.09	1.1 (0.8, 1.5)	0.42
**TNFR-2**									
	Per doubling	1.5 (1.3, 1.8)	<0.01	1.4 (1.2, 1.7)	<0.01	1.4 (1.1, 1.6)	<0.01	1.2 (1.0, 1.5)	0.07
	Quartile 1	(reference)		(reference)		(reference)		(reference)	
	Quartile 2	1.0 (0.8, 1.3)	0.98	1.0 (0.7, 1.3)	0.79	0.9 (0.7, 1.2)	0.70	0.9 (0.7, 1.2)	0.67
	Quartile 3	1.1 (0.8, 1.4)	0.55	1.0 (0.8, 1.3)	0.91	1.0 (0.8, 1.3)	0.93	1.0 (0.7, 1.3)	0.77
	Quartile 4	1.8 (1.4, 2.3)	<0.01	1.6 (1.3, 2.1)	<0.01	1.5 (1.2, 2.0)	<0.01	1.4 (1.0, 1.8)	0.03

**KIM-1**									
	Per doubling	1.3 (1.2, 1.4)	<0.01	1.2 (1.1, 1.3)	<0.01	1.1 (1.0, 1.2)	0.01	1.0 (0.9, 1.1)	0.56
	Quartile 1	(reference)		(reference)		(reference)		(reference)	
	Quartile 2	1.0 (0.8, 1.3)	0.75	1.0 (0.7, 1.2)	0.70	0.9 (0.7, 1.2)	0.58	0.9 (0.7, 1.1)	0.35
	Quartile 3	1.1 (0.9, 1.4)	0.38	1.0 (0.8, 1.3)	0.98	1.0 (0.8, 1.3)	0.85	0.9 (0.7, 1.2)	0.38
	Quartile 4	1.5 (1.2, 1.9)	<0.01	1.2 (0.9, 1.5)	0.17	1.1 (0.9, 1.4)	0.41	0.9 (0.7, 1.1)	0.31

NOTE: Models are adjusted for the following covariates. Model 1: baseline biomarker (TNFR-1, TNFR-2, or KIM-1 in quartiles or continuous), age, sex, race, and randomised treatment. Model 2: covariates of model 1 + history of CVD, HbA_{1c}, current smoking, systolic and diastolic BP, BMI, and LDL cholesterol. Model 3: covariates of model 2 + baseline eGFR. Model 4: covariates of model 3 + log transformed baseline UACR.
KIM-1, kidney injury molecule-1; TNFR-1, tumour necrosis factor receptor-1; TNFR-2, tumour necrosis factor receptor-2; UACR, urine albumin/creatinine ratio.

ESM Table 2. Associations of the quartiles of TNFR-1, TNFR-2, and KIM-1 with the heart failure outcome

**Biomarker**		**Model 1**		**Model 2**		**Model 3**		**Model 4**	
		**HR (95% CI)**	***p* value**	**HR (95% CI)**	***p* value**	**HR (95% CI)**	***p* value**	**HR (95% CI)**	***p* value**
**TNFR-1**									
	Per doubling	3.2 (2.2, 4.6)	<0.01	2.0 (1.4, 2.9)	<0.01	1.7 (1.0, 2.7)	0.03	1.1 (0.7, 1.8)	0.60
	Quartile 1	(reference)		(reference)		(reference)		(reference)	
	Quartile 2	1.3 (0.7, 2.6)	0.41	1.1 (0.5, 2.1)	0.87	1.0 (0.5, 2.0)	0.97	1.0 (0.5, 2.0)	0.95
	Quartile 3	2.3 (1.2, 4.3)	0.01	1.6 (0.9, 3.1)	0.13	1.5 (0.8, 2.8)	0.24	1.3 (0.7, 2.5)	0.39
	Quartile 4	3.9 (2.2, 7.1)	<0.01	2.3 (1.3, 4.3)	0.01	1.9 (1.0, 3.7)	0.06	1.5 (0.7, 2.9)	0.26
**TNFR-2**									
	Per doubling	2.2 (1.7, 2.9)	<0.01	1.7 (1.2, 2.4)	<0.01	1.4 (0.9, 2.1)	0.12	1.0 (0.7, 1.6)	0.89
	Quartile 1	(reference)		(reference)		(reference)		(reference)	
	Quartile 2	1.6 (0.9, 2.8)	0.14	1.2 (0.7, 2.2)	0.48	1.2 (0.6, 2.2)	0.61	1.2 (0.6, 2.1)	0.64
	Quartile 3	1.3 (0.7, 2.4)	0.41	0.9 (0.5, 1.8)	0.87	0.9 (0.5, 1.6)	0.64	0.8 (0.4, 1.5)	0.52
	Quartile 4	3.1 (1.8, 5.3)	<0.01	1.9 (1.1, 3.3)	0.03	1.5 (0.8, 2.7)	0.21	1.1 (0.6, 2.1)	0.70

**KIM-1**									
Per doubling	1.6 (1.3, 1.9)	<0.01	1.4 (1.2, 1.7)	<0.01	1.4 (1.1, 1.6)	<0.01	1.1 (0.9, 1.3)	0.40
Quartile 1	(reference)		(reference)		(reference)		(reference)	
Quartile 2	0.9 (0.5, 1.8)	0.85	0.8 (0.4, 1.5)	0.43	0.7 (0.4, 1.4)	0.35	0.7 (0.3, 1.3)	0.21
Quartile 3	1.8 (1.0, 3.2)	0.03	1.5 (0.9, 2.6)	0.16	1.4 (0.8, 2.5)	0.22	1.2 (0.7, 2.1)	0.57
Quartile 4	2.7 (1.6, 4.5)	<0.01	1.8 (1.1, 3.2)	0.03	1.6 (0.9, 2.8)	0.10	1.0 (0.5, 1.8)	0.96

NOTE: Models are adjusted for the following covariates. Model 1: age, sex, race, and randomised treatment. Model 2: covariates of model 1 + history of CVD, HbA_{1c}, current smoking, systolic and diastolic BP, BMI, and LDL cholesterol. Model 3: covariates of model 2 + baseline eGFR. Model 4: covariates of model 3 + log transformed baseline UACR.

KIM-1, kidney injury molecule-1; TNFR-1, tumour necrosis factor receptor-1; TNFR-2, tumour necrosis factor receptor-2; UACR, urine albumin/creatinine ratio.

ESM Table 3. C-statistics of the Cox-proportional hazard regression models used to assess the association between doubling in biomarker with outcomes with and without biomarker

	**Full model without biomarker**	**TNFR-1**	**TNFR-2**	**KIM-1**
		**Kidney outcome**		
C-statistic (95% CI)	0.789 (0.747, 0.830)	0.807 (0.769, 0.845)	0.813 (0.775, 0.850)	0.797 (0.757, 0.838)
IDI (SD); *p* value	-	0.011 (0.004); *p*<0.01	0.013 (0.004); *p*<0.01	0.012 (0.004); *p*<0.01
NRI (SD); *p* value	-	0.290 (0.087); *p*<0.01	0.411 (0.087); *p*<0.01	0.2544 (0.087); *p*<0.01
		**CV outcome**		
C-statistic (95% CI)	0.680 (0.656, 0.704)	0.681 (0.657, 0.705)	0.682 (0.658, 0.706)	0.680 (0.657, 0.704)
IDI (SD); *p* value	-	0.001 (0.001); *p*=0.09	0.001 (0.001); *p*=0.10	0.000 (0.000); *p*=0.80
NRI (SD); *p* value	-	0.093 (0.047); *p*=0.04	0.059 (0.047); *p*=0.21	0.026 (0.047); *p*=0.57
		**HF outcome**		
C-statistic (95% CI)	0.799 (0.765, 0.833)	0.798 (0.764, 0.833)	0.799 (0.765, 0.833)	0.800 (0.766, 0.834)
IDI (SD); *p* value	-	0.001 (0.000); *p*=0.10	0.000 (0.000); *p*=0.30	0.000 (0.001); *p*=0.99)
NRI (SD); *p* value	-	-0.035 (0.090); *p*=0.99	-0.026 (0.090); *p*=0.99	0.057 (0.090); *p*=0.53

NOTE: The full model without biomarker was adjusted for the following covariates: age, sex, race, and randomised treatment, history of CVD, HbA_{1c}, current smoking, systolic and diastolic BP, BMI, and LDL cholesterol, baseline eGFR, log transformed baseline urine albumin/creatinine ratio.

ESM Table 4. Time dependent AUC values of baseline biomarkers during 6 years follow-up for the kidney, CV, and HF outcome

	C-statistic
	Kidney outcome
TNFR-1	0.710
TNFR-2	0.727
KIM-1	0.730
	CV outcome
TNFR-1	0.577
TNFR-2	0.574
KIM-1	0.574
	HF outcome
TNFR-1	0.662
TNFR-2	0.627
KIM-1	0.641

Note: Time dependent AUC values are obtained from unadjusted models

ESM Table 5. Associations of the quartiles of each change in TNFR-1, TNFR-2, and KIM-1 from baseline to year 1 with the composite cardiovascular outcome

Biomarker		Model 1		Model 2		Model 3		Model 4		Model 5	
	**Median change**	**HR (95% CI)**	***p* value**	**HR (95% CI)**	***p* value**	**HR (95% CI)**	**p value**	**HR (95% CI)**	***p* value**	**HR (95% CI)**	***p* value**
**TNFR-1**											
Per 10% reduction		1.0 (0.9, 1.1)	0.54	1.0 (0.9, 1.1)	0.83	1.0 (0.9, 1.1)	0.91	1.0 (0.9, 1.1)	0.74	1.0 (1.0, 1.1)	0.47
Quartile 1	−11.7	1.0 (0.7, 1.3)	0.99	1.0 (0.8, 1.4)	0.89	1.0 (0.7, 1.3)	1.00	1.0 (0.8, 1.4)	0.88	1.0 (0.8, 1.4)	0.91
Quartile 2	−0.6	(reference)		(reference)		(reference)		(reference)		(reference)	
Quartile 3	8.2	0.9 (0.7, 1.2)	0.46	0.9 (0.6, 1.2)	0.39	0.9 (0.7, 1.2)	0.42	0.9 (0.6, 1.2)	0.34	0.9 (0.6, 1.2)	0.31
Quartile 4	21.1	1.0 (0.7, 1.4)	0.99	1.0 (0.7, 1.3)	0.79	0.9 (0.7, 1.3)	0.62	0.9 (0.6, 1.2)	0.41	0.8 (0.6, 1.1)	0.21
**TNFR-2**											
Per 10% reduction		1.0 (0.9, 1.0)	0.63	1.0 (0.9, 1.1)	0.96	1.0 (0.9, 1.1)	0.83	1.0 (0.9, 1.1)	0.85	1.0 (0.9, 1.1)	0.77
Quartile 1	−12.8	1.0 (0.7, 1.3)	0.96	1.0 (0.7, 1.4)	0.97	1.0 (0.7, 1.3)	0.89	1.0 (0.7, 1.3)	0.95	1.0 (0.8, 1.4)	0.93
Quartile 2	−2.2	(reference)		(reference)		(reference)		(reference)		(reference)	
Quartile 3	6.2	0.9 (0.7, 1.2)	0.43	0.9 (0.6, 1.2)	0.34	0.9 (0.6, 1.2)	0.37	0.8 (0.6, 1.1)	0.28	0.9 (0.6, 1.2)	0.32
Quartile 4	20.0	1.1 (0.8, 1.5)	0.44	1.1 (0.8, 1.4)	0.70	1.1 (0.8, 1.4)	0.68	1.0 (0.7, 1.4)	0.97	1.0 (0.7, 1.4)	0.99

**KIM-1**											
Per 10% reduction		1.0 (0.9, 1.0)	0.02	1.0 (0.9, 1.0)	0.02	1.0 (0.9, 1.0)	0.19	1.0 (0.9, 1.0)	0.22	1.0 (1.0, 1.0)	0.71
Quartile 1	−34.0	0.7 (0.5, 1.0)	0.03	0.7 (0.5, 1.0)	0.03	0.7 (0.5, 1.0)	0.06	0.7 (0.5, 1.0)	0.06	0.8 (0.6, 1.0)	0.10
Quartile 2	−13.3	(reference)		(reference)		(reference)		(reference)		(reference)	
Quartile 3	2.9	1.0 (0.7, 1.3)	0.83	1.0 (0.7, 1.3)	0.84	0.9 (0.7, 1.2)	0.60	0.9 (0.7, 1.2)	0.61	0.9 (0.7, 1.2)	0.57
Quartile 4	33.1	1.2 (0.9, 1.6)	0.29	1.2 (0.9, 1.6)	0.32	1.1 (0.8, 1.4)	0.64	1.1 (0.8, 1.4)	0.70	1.0 (0.7, 1.3)	0.77

NOTE: Models are adjusted for the following covariates. Model 1: baseline biomarker (TNFR-1, TNFR-2, or KIM-1), age, sex, race, and randomised treatment. Model 2: covariates of model 1 + change in eGFR from baseline to year 1 and baseline eGFR. Model 3: covariates of model 1 + change in UACR from baseline to year 1 and baseline UACR. Model 4: covariates of model 1 + change in eGFR and UACR from baseline to year 1 and baseline eGFR and UACR. Model 5: covariates of model 1 + history of CVD, current smoking, HbA_{1c}, systolic and diastolic BP, BMI, LDL cholesterol, eGFR, baseline UACR, and change in eGFR, UACR, systolic BP, BMI, and HbA_{1c} from baseline to year 1.

KIM-1, kidney injury molecule-1; TNFR-1, tumour necrosis factor receptor-1; TNFR-2, tumour necrosis factor receptor-2; UACR, urine albumin/creatinine ratio.

ESM Table 6. Associations of the quartiles of each change in TNFR-1, TNFR-2, and KIM-1 from baseline to year 1 with the composite heart failure outcome

**Biomarker**		**Model 1**		**Model 2**		**Model 3**		**Model 4**		**Model 5**	
	**Median change**	**HR (95% CI)**	***p* value**	**HR (95% CI)**	***p* value**	**HR (95% CI)**	***p* value**	**HR (95% CI)**	***p* value**	**HR (95% CI)**	***p* value**
**TNFR-1**											
Per 10% reduction		0.9 (0.8, 1.0)	0.14	0.9 (0.8, 1.1)	0.26	0.9 (0.8, 1.1)	0.38	1.0 (0.8, 1.1)	0.60	1.0 (0.9, 1.2)	0.89
Quartile 1	−12.4	1.7 (0.9, 3.3)	0.10	1.8 (0.9, 3.4)	0.08	1.7 (0.9, 3.3)	0.09	1.8 (0.9, 3.4)	0.08	1.9 (1.0, 3.6)	0.06
Quartile 2	−0.4	(reference)		(reference)		(reference)		(reference)		(reference)	
Quartile 3	8.1	1.9 (1.0, 3.6)	0.06	1.8 (0.9, 3.6)	0.07	1.8 (1.0, 3.6)	0.07	1.8 (0.9, 3.5)	0.08	1.8 (0.9, 3.5)	0.09
Quartile 4	19.2	2.3 (1.2, 4.4)	0.01	2.2 (1.2, 4.3)	0.02	2.0 (1.1, 3.8)	0.03	1.9 (1.0, 3.7)	0.05	1.7 (0.9, 3.2)	0.12
**TNFR-2**											
Per 10% reduction		0.9 (0.9, 1.0)	0.07	0.9 (0.9, 1.0)	0.18	0.9 (0.9, 1.0)	0.08	0.9 (0.9, 1.0)	0.20	1.0 (0.8, 1.1)	0.39
Quartile 1	−13.7	2.1 (1.1, 4.0)	0.03	2.1 (1.1, 4.0)	0.03	2.0 (1.0, 3.9)	0.04	2.0 (1.1, 4.0)	0.03	2.2 (1.1, 4.4)	0.02
Quartile 2	−2.1	(reference)		(reference)		(reference)		(reference)		(reference)	
Quartile 3	6.0	1.8 (0.9, 3.5)	0.09	1.7 (0.9, 3.4)	0.12	1.8 (0.9, 3.6)	0.08	1.7 (0.9, 3.4)	0.11	1.8 (0.9, 3.5)	0.11
Quartile 4	18.2	2.4 (1.2, 4.6)	0.01	2.1 (1.1, 4.2)	0.03	2.2 (1.1, 4.2)	0.02	2.0 (1.0, 3.9)	0.05	1.9 (0.9, 3.7)	0.07

**KIM-1**											
Per 10% reduction		0.9 (0.9, 1.0)	0.05	0.9 (0.9, 1.0)	0.06	1.0 (0.9, 1.0)	0.28	1.0 (0.9, 1.0)	0.38	1.0 (0.9, 1.0)	0.20
Quartile 1	−41.3	0.6 (0.3, 1.2)	0.14	0.6 (0.4, 1.2)	0.14	0.7 (0.4, 1.3)	0.23	0.7 (0.4, 1.3)	0.24	0.7 (0.4, 1.2)	0.18
Quartile 2	−14.3	(reference)		(reference)		(reference)		(reference)		(reference)	
Quartile 3	3.0	1.1 (0.6, 1.8)	0.81	1.1 (0.6, 1.8)	0.82	1.0 (0.6, 1.7)	1.00	1.0 (0.6, 1.7)	0.98	1.1 (0.6, 1.9)	0.80
Quartile 4	28.8	1.0 (0.6, 1.8)	0.89	1.0 (0.6, 1.8)	1.00	0.9 (0.5, 1.6)	0.70	0.8 (0.5, 1.5)	0.56	0.9 (0.5, 1.6)	0.74

NOTE: Models are adjusted for the following covariates. Model 1: baseline biomarker (TNFR-1, TNFR-2, or KIM-1), age, sex, race, and randomised treatment. Model 2: covariates of model 1 + change in eGFR from baseline to year 1 and baseline eGFR. Model 3: covariates of model 1 + change in UACR from baseline to year 1 and baseline UACR. Model 4: covariates of model 1 + change in eGFR and UACR from baseline to year 1 and baseline eGFR and UACR. Model 5: covariates of model 1 + history of CVD, current smoking, HbA_{1c}, systolic and diastolic BP, BMI, LDL cholesterol, eGFR, baseline UACR, and change in eGFR, UACR, systolic BP, BMI, and HbA_{1c} from baseline to year 1.

KIM-1, kidney injury molecule-1; TNFR-1, tumour necrosis factor receptor-1; TNFR-2, tumour necrosis factor receptor-2; UACR, urine albumin/creatinine ratio.

ESM Table 7. Associations within the canagliflozin group of quartiles of 1-year change from baseline in TNFR-1, TNFR-2, and KIM-1 with the composite kidney, cardiovascular, and heart failure outcome in the fully adjusted model

**Biomarker**	**Kidney outcome**		**Cardiovascular outcome**		**Heart failure outcome**	
	**HR (95% CI)**	***p* value**	**HR (95% CI)**	***p* value**	**HR (95% CI)**	***p* value**
**TNFR-1**						
Per 10% reduction	0.90 (0.74, 1.11)	0.34	1.05 (0.97, 1.14)	0.24	1.04 (0.85, 1.26)	0.72
Quartile 1	0.70 (0.30, 1.63)	0.41	1.01 (0.70, 1.44)	0.98	1.93 (0.88, 4.21)	0.10
Quartile 2	(reference)		(reference)		(reference)	
Quartile 3	0.97 (0.43, 2.2)	0.95	0.84 (0.58, 1.23)	0.37	1.39 (0.59, 3.26)	0.45
Quartile 4	1.38 (0.68, 2.78)	0.38	0.71 (0.48, 1.05)	0.08	1.42 (0.63, 3.21)	0.40
**TNFR-2**						
Per 10% reduction	0.92 (0.87, 0.98)	0.01	1.04 (0.95, 1.14)	0.35	0.97 (0.82, 1.16)	0.75
Quartile 1	0.57 (0.23, 1.44)	0.23	0.93 (0.65, 1.34)	0.71	2.27 (1.00, 5.17)	0.05
Quartile 2	(reference)		(reference)		(reference)	
Quartile 3	1.15 (0.50, 2.65)	0.74	0.86 (0.60, 1.25)	0.44	1.19 (0.47, 3.03)	0.72
Quartile 4	2.07 (0.99, 4.33)	0.06	0.80 (0.55, 1.17)	0.25	2.02 (0.85, 4.78)	0.11

**KIM-1**						
Per 10% reduction	0.94 (0.89, 1.02)	0.16	1.00 (0.95, 1.05)	0.99	0.96 (0.87, 1.06)	0.43
Quartile 1	1.23 (0.58, 2.59)	0.59	0.81 (0.56, 1.16)	0.25	0.73 (0.36, 1.48)	0.38
Quartile 2	(reference)		(reference)		(reference)	
Quartile 3	1.63 (0.74, 3.58)	0.22	0.92 (0.64, 1.32)	0.66	1.15 (0.58, 2.28)	0.69
Quartile 4	1.06 (0.42, 2.65)	0.91	0.92 (0.62, 1.36)	0.67	0.56 (0.22, 1.46)	0.24

NOTE: Interaction *p* values for TNFR-1 and TNFR-2 with treatment assignment for the kidney outcome were not statically significant ($p = 0.60$ and $p = 0.20$) indicating that the association between 1-year changes from baseline in TNFR-1 and TNFR-2 were consistent in the canagliflozin and placebo group. Models are adjusted for the following covariates. Model 1: baseline biomarker (TNFR-1, TNFR-2, or KIM-1), age, sex, race, and randomised treatment. Model 2: covariates of model 1 + change in eGFR from baseline to year 1 and baseline eGFR. Model 3: covariates of model 1 + change in UACR from baseline to year 1 and baseline UACR. Model 4: covariates of model 1 + change in eGFR and UACR from baseline to year 1 and baseline eGFR and UACR. Model 5: covariates of model 1 + history of CVD, current smoking, HbA_{1c}, systolic and diastolic BP, BMI, LDL cholesterol, eGFR, baseline UACR, and change in eGFR, UACR, systolic BP, BMI, and HbA_{1c} from baseline to year 1.

KIM-1, kidney injury molecule-1; TNFR-1, tumour necrosis factor receptor-1; TNFR-2, tumour necrosis factor receptor-2; UACR, urine albumin/creatinine ratio.

ESM Table 8. Proportion of treatment effects explained by changes in TNFR-1, TNFR-2, or KIM-1 from baseline to 1-year follow-up

	Unadjusted HR (95% CI)	Adjusted HR (95% CI)	Proportion explained (%)
Kidney outcome			
TNFR-1	0.56 (0.38, 0.81)	0.53 (0.36, 0.77)	−6.8
TNFR-2	0.56 (0.38, 0.81)	0.50 (0.34, 0.74)	−13.6
KIM-1	0.56 (0.38, 0.81)	0.58 (0.40, 0.86)	4.5
Cardiovascular outcome			
TNFR-1	0.92 (0.73, 1.15)	0.91 (0.73, 1.14)	−12.5
TNFR-2	0.92 (0.73, 1.15)	0.91 (0.73, 1.14)	−12.5
KIM-1	0.92 (0.73, 1.15)	0.97 (0.77, 1.22)	62.5
Heart failure outcome			
TNFR-1	0.82 (0.54, 1.25)	0.80 (0.53, 1.22)	−11.1
TNFR-2	0.82 (0.54, 1.25)	0.79 (0.52, 1.20)	−16.7
KIM-1	0.82 (0.54, 1.25)	0.89 (0.58, 1.37)	38.9

KIM-1, kidney injury molecule-1; TNFR-1, TNF receptor-1; TNFR-2, TNF receptor-2.

ESM Fig. 1 Flow diagram of study design and analysis.

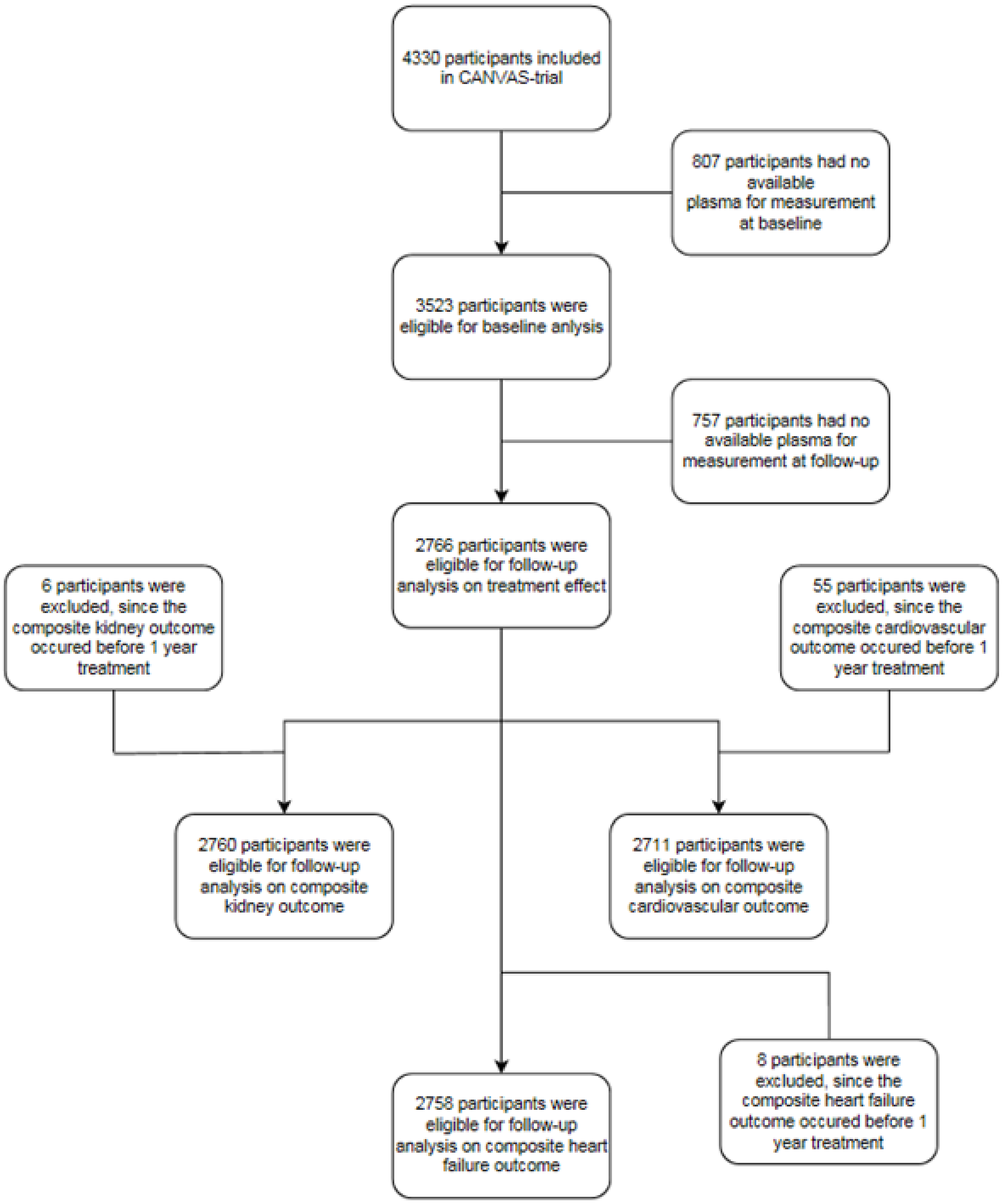

ESM Fig. 2 Pearson correlation test of each baseline biomarker with covariates used in the assessment of the association of each baseline biomarker with kidney and cardiovascular outcomes.

−0.6	−0.4	−0.2	0	0.2	0.4	0.6

	TNFR-1	TNFR-2	KIM-1
Age (years)	0.24	0.18	0.17
Sex (male/female)	−0.01	−0.04	−0.01
Race	0.01	−0.06	0.07
Treatment with canagliflozin (yes/no)	0.00	−0.01	−0.01
History of CVD	0.05	0.06	0.03
Current smoking	−0.10	−0.08	0.01
HbA_{1c}	0.06	0.07	0.14
Systolic BP	0.08	0.04	0.18
Diastolic BP	−0.13	−0.13	0.02
BMI	0.17	0.12	0.05
LDL cholesterol	−0.06	−0.07	0.10
eGFR	−0.54	−0.45	−0.26
UACR	0.29	0.26	0.47

KIM-1, kidney injury molecule-1; TNFR-1, tumour necrosis factor receptor-1; TNFR-2, tumour necrosis factor receptor-2; UACR, urine albumin/creatinine ratio.

ESM Fig. 3 Associations of the doubling in each baseline biomarker with the cardiovascular outcome by subpopulations.

Subgroup	Number of patients/number of patients with an event (N/n)	HR (95% CI)	*p* for heterogeneity
TNFR-1			
Overall	3523/548	1.3 (1.0, 1.6)	
Treatment			0.31
Placebo	1181/193	1.3 (0.9, 2.0)	
Canagliflozin	2342/355	1.2 (0.9, 1.7)	
Age			0.07
<65 years	2071/287	1.1 (0.8, 1.6)	
≥65 years	1452/261	1.4 (1.0, 2.0)	
Sex			0.16
Male	2365/393	1.3 (1.0, 1.7)	
Female	1158/155	1.4 (0.9, 2.1)	
UACR			0.20
<3.39 mg/mmol	2547/341	1.1 (0.8, 1.5)	
≥3.39 mg/mmol	976/207	1.4 (1.0, 2.1)	
eGFR			0.31
≥60 ml min^{-1} [1.73 m]$^{-2}$	2944/423	1.2 (0.9, 1.6)	
<60 ml min^{-1} [1.73 m]$^{-2}$	579/125	1.7 (1.0, 2.8)	
CVD history			0.29
No	1426/128	1.5 (0.9, 2.5)	
Yes	2097/420	1.2 (0.9, 1.6)	
TNFR-2			
Overall	3523/548	1.2 (1.0, 1.5)	
Treatment			0.16
Placebo	1181/193	1.4 (1.0, 2.1)	
Canagliflozin	2342/355	1.1 (0.9, 1.4)	
Age			0.02
<65 years	2071/287	1.0 (0.8, 1.3)	
≥65 years	1452/261	1.4 (1.0, 2.0)	
Sex			0.27
Male	2365/393	1.2 (1.0, 1.6)	
Female	1158/155	1.2 (0.9, 1.8)	
UACR			0.81
<3.39 mg/mmol	2547/341	1.2 (0.9, 1.6)	
≥3.39 mg/mmol	976/207	1.1 (0.8, 1.6)	
eGFR			0.07
≥60 ml min^{-1} [1.73 m]$^{-2}$	2944/423	1.1 (0.9, 1.4)	
<60 ml min^{-1} [1.73 m]$^{-2}$	579/125	1.9 (1.2, 3.1)	
CVD history			0.80
No	1426/128	1.0 (0.7, 1.5)	
Yes	2097/420	1.3 (1.0, 1.6)	
KIM-1			
Overall	3523/548	1.0 (0.9, 1.1)	
Treatment			0.10
Placebo	1181/193	1.1 (0.9, 1.3)	
Canagliflozin	2342/355	1.0 (0.9, 1.1)	
Age			0.19
<65 years	2071/287	1.0 (0.8, 1.1)	
≥65 years	1452/261	1.1 (1.0, 1.3)	
Sex			0.52
Male	2365/393	1.1 (0.9, 1.2)	
Female	1158/155	0.9 (0.8, 1.1)	
UACR			0.84
<3.39 mg/mmol	2547/341	1.0 (0.9, 1.2)	
≥3.39 mg/mmol	976/207	1.0 (0.9, 1.2)	
eGFR			0.14
≥60 ml min^{-1} [1.73 m]$^{-2}$	2944/423	1.0 (0.9, 1.1)	
<60 ml min^{-1} [1.73 m]$^{-2}$	579/125	1.2 (1.0, 1.5)	
CVD history			<0.01
No	1426/128	1.3 (1.1, 1.6)	
Yes	2097/420	1.0 (0.8, 1.1)	

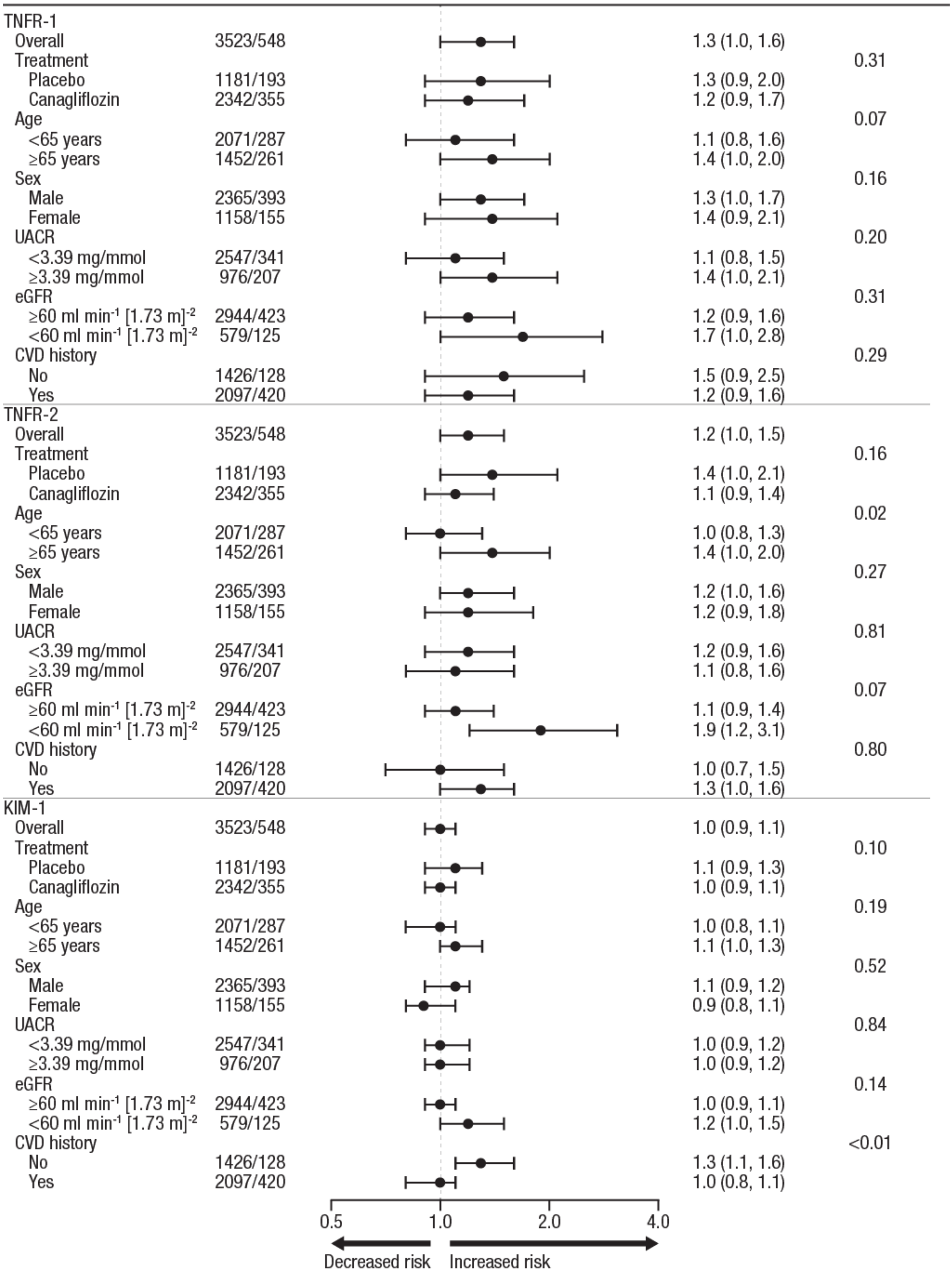

NOTE: Models are adjusted for the following covariates: age, sex, race, and randomised treatment, history of CVD, HbA_{1c}, current smoking, systolic and diastolic BP, BMI, and LDL cholesterol, baseline eGFR, log transformed baseline urine albumin/creatinine ratio.

KIM-1, kidney injury molecule-1; TNFR-1, tumour necrosis factor receptor-1; TNFR-2, tumour necrosis factor receptor-2; UACR, urine albumin/creatinine ratio.

ESM Fig. 4 Associations of the doubling in each baseline biomarker with the heart failure outcome by subpopulations.

Subgroup	Number of patients/number of patients with an event (N/n)	HR (95% CI)	*p* for heterogeneity
TNFR-1			
Overall	3523/128	1.1 (0.7, 1.8)	
Treatment			0.52
Placebo	1181/48	1.1 (0.5, 2.5)	
Canagliflozin	2342/80	1.2 (0.6, 2.2)	
Age			0.01
<65 years	2071/56	2.3 (1.1, 4.9)	
≥65 years	1452/72	0.7 (0.4, 1.4)	
Sex			0.39
Male	2365/97	1.2 (0.7, 2.2)	
Female	1158/31	0.8 (0.3, 1.9)	
UACR			0.01
<3.39 mg/mmol	2547/67	0.5 (0.3, 1.0)	
≥3.39 mg/mmol	976/61	2.3 (1.1, 4.8)	
eGFR			0.70
≥60 ml min^{-1} [1.73 m]$^{-2}$	2944/88	1.1 (0.6, 2.0)	
<60 ml min^{-1} [1.73 m]$^{-2}$	579/40	0.9 (0.4, 2.3)	
CVD history			0.80
No	1426/29	0.8 (0.3, 2.3)	
Yes	2097/99	1.2 (0.7, 2.2)	
TNFR-2			
Overall	3523/128	1.0 (0.7, 1.6)	
Treatment			0.25
Placebo	1181/48	0.9 (0.4, 1.8)	
Canagliflozin	2342/80	1.2 (0.7, 2.0)	
Age			0.03
<65 years	2071/56	1.5 (0.8, 2.9)	
≥65 years	1452/72	0.8 (0.5, 1.4)	
Sex			0.15
Male	2365/97	1.0 (0.6, 1.6)	
Female	1158/31	1.1 (0.4, 2.8)	
UACR			0.66
<3.39 mg/mmol	2547/67	0.9 (0.5, 1.8)	
≥3.39 mg/mmol	976/61	1.0 (0.5, 1.9)	
eGFR			0.46
≥60 ml min^{-1} [1.73 m]$^{-2}$	2944/88	0.9 (0.6, 1.6)	
<60 ml min^{-1} [1.73 m]$^{-2}$	579/40	1.0 (0.4, 2.6)	
CVD history			0.63
No	1426/29	0.5 (0.2, 1.3)	
Yes	2097/99	1.3 (0.8, 2.1)	
KIM-1			
Overall	3523/128	1.1 (0.9, 1.3)	
Treatment			0.45
Placebo	1181/48	1.1 (0.8, 1.5)	
Canagliflozin	2342/80	1.1 (0.8, 1.4)	
Age			0.53
<65 years	2071/56	0.9 (0.7, 1.3)	
≥65 years	1452/72	1.2 (0.9, 1.6)	
Sex			0.77
Male	2365/97	1.1 (0.9, 1.4)	
Female	1158/31	1.0 (0.7, 1.6)	
UACR			0.76
<3.39 mg/mmol	2547/67	1.2 (0.9, 1.6)	
≥3.39 mg/mmol	976/61	0.9 (0.7, 1.2)	
eGFR			0.88
≥60 ml min^{-1} [1.73 m]$^{-2}$	2944/88	1.0 (0.8, 1.4)	
<60 ml min^{-1} [1.73 m]$^{-2}$	579/40	1.0 (0.7, 1.5)	
CVD history			0.09
No	1426/29	1.5 (0.9, 2.3)	
Yes	2097/99	1.0 (0.8, 1.3)	

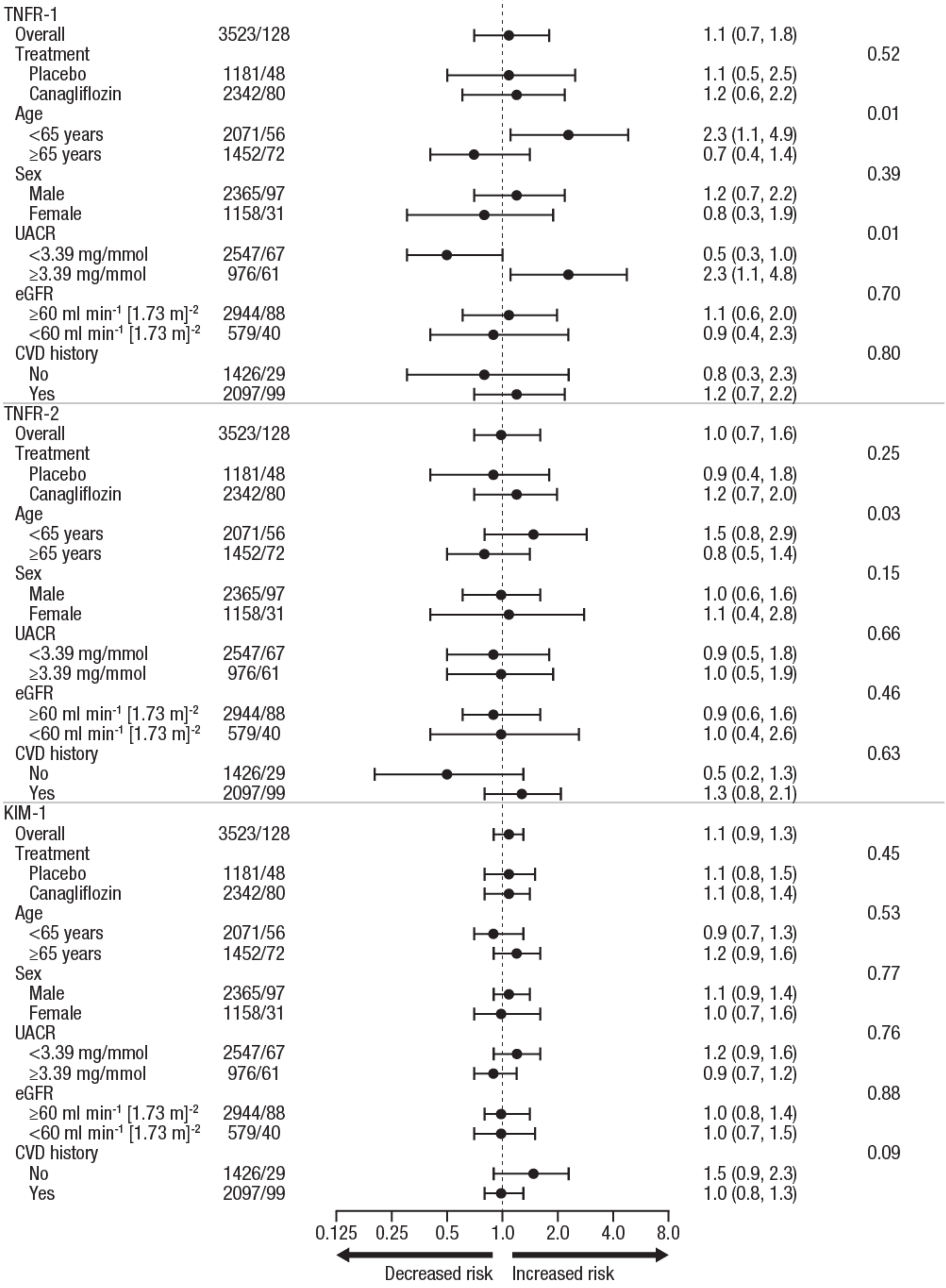

NOTE: Models are adjusted for the following covariates: age, sex, race, and randomised treatment, history of CVD, HbA_{1c}, current smoking, systolic and diastolic BP, BMI, and LDL cholesterol, baseline eGFR, log transformed baseline urine albumin/creatinine ratio.

When UACR was fitted as dichotomous variable the *p* value for heterogeneity between UACR-categories and TNFR-1 was 0.02.

KIM-1, kidney injury molecule-1; TNFR-1, tumour necrosis factor receptor-1; TNFR-2, tumour necrosis factor receptor-2; UACR, urine albumin/creatinine ratio.

ESM Fig. 5 Forest plot of the effect of canagliflozin on heart failure outcome by baseline TNFR-1, TNFR-2, and KIM-1 divided in tertiles.

Tertile	Number of patients with an event (n/N) Canagliflozin	Placebo	HR (95% CI)	*p* for heterogeneity
TNFR-1				0.15
Tertile 1	11/781	8/395	0.69 (0.28, 1.72)	
Tertile 2	22/773	21/401	0.51 (0.28, 0.93)	
Tertile 3	47/788	19/385	1.14 (0.67, 1.95)	
TNFR-2				0.41
Tertile 1	13/398	15/778	0.58 (0.28, 1.23)	
Tertile 2	14/384	20/789	0.68 (0.34, 1.34)	
Tertile 3	21/399	45/775	1.02 (0.61, 1.72)	
KIM-1				0.85
Tertile 1	10/395	14/803	0.67 (0.30, 1.52)	
Tertile 2	15/378	25/774	0.80 (0.42, 1.52)	
Tertile 3	23/408	41/765	0.88 (0.53, 1.47)	

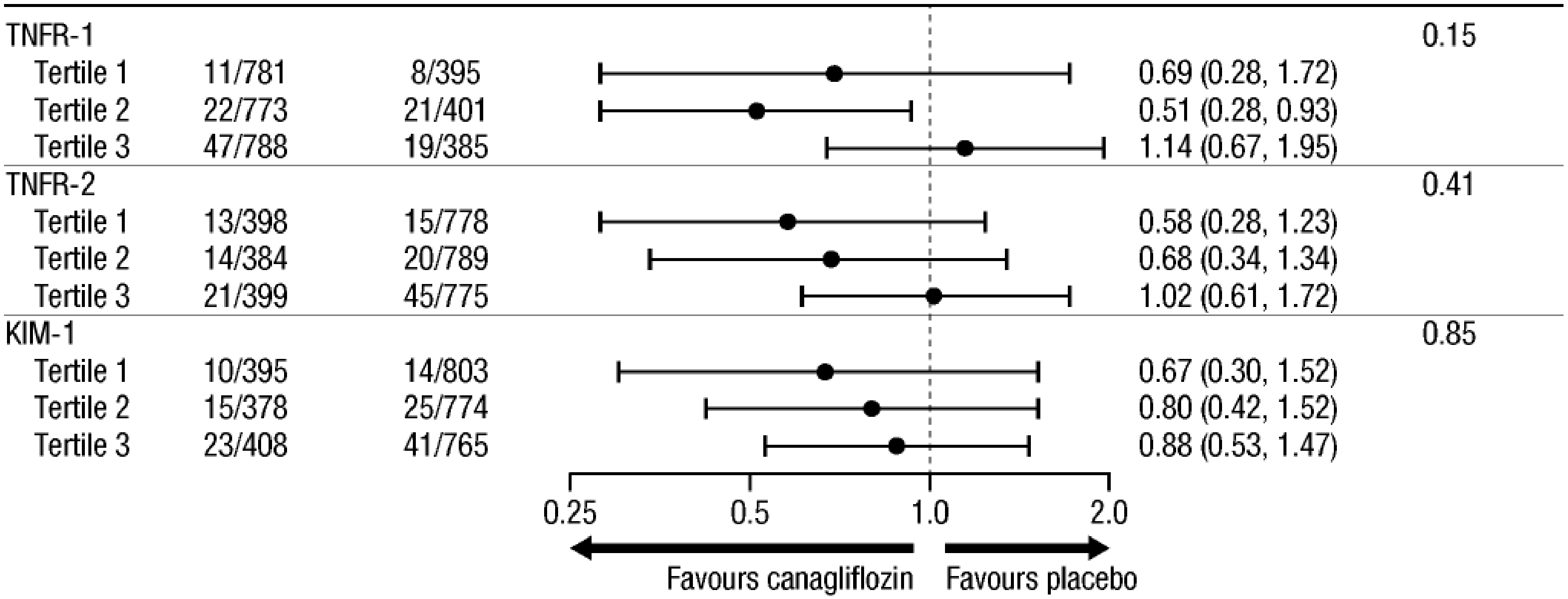

KIM-1, kidney injury molecule-1; TNFR-1, tumour necrosis factor receptor-1; TNFR-2, tumour necrosis factor receptor-2.

ESM Fig. 6 Pearson correlation test of each change in biomarker from baseline to year 1 with covariates used in the assessment of the association of each change in biomarker from baseline to year 1 with kidney and cardiovascular outcomes.

−0.6 −0.4 −0.2 0 0.2 0.4 0.6

	Change in TNFR-1	Change in TNFR-2	Change in KIM-1
Baseline biomarker (TNFR-1, TNFR-2, or KIM-1)	−0.13	−0.15	−0.30
Treatment with canagliflozin (yes/no)	−0.02	0.01	−0.22
Age	0.05	0.06	−0.05
Sex	−0.04	−0.01	0.04
Race	0.00	0.02	0.04
History of CVD	−0.01	−0.02	0.03
Current smoking	−0.02	−0.03	0.03
HbA_{1c}	0.05	0.03	0.15
Systolic BP	−0.07	−0.06	0.06
Diastolic BP	−0.08	−0.09	0.05
BMI	0.02	0.01	0.02
LDL cholesterol	0.05	0.02	0.10
eGFR	−0.12	−0.11	0.08
Change in UACR from baseline	−0.05	−0.08	0.20
Change in systolic BP	−0.13	−0.13	0.13
Change in diastolic BP	0.07	0.02	0.10
Change in HbA_{1c}	0.04	0.03	0.23
Baseline UACR	0.08	0.06	−0.09
Change in eGFR	−0.23	−0.17	0.02

KIM-1, kidney injury molecule-1; TNFR-1, tumour necrosis factor receptor-1; TNFR-2, tumour necrosis factor receptor-2; UACR, urine albumin/creatinine ratio
